# Two-photon imaging of excitatory and inhibitory neural response to infrared neural stimulation

**DOI:** 10.1117/1.NPh.11.2.025003

**Published:** 2024-05-24

**Authors:** Peng Fu, Yin Liu, Liang Zhu, Mengqi Wang, Yuan Yu, Fen Yang, Weijie Zhang, Hequn Zhang, Shy Shoham, Anna Wang Roe, Wang Xi

**Affiliations:** a Second Affiliated Hospital, Zhejiang University, Interdisciplinary Institute of Neuroscience and Technology, School of Medicine, Hangzhou, China; b KU Leuven Medical School, Laboratory for Neuro- and Psychophysiology, Department of Neurosciences, Leuven, Belgium; c Zhejiang University, College of Biomedical Engineering and Instrument Science, Key Laboratory of Biomedical Engineering of Ministry of Education, Hangzhou, China; d NYU Langone Health, Department of Ophthalmology and Tech4Health and Neuroscience Institutes, New York, New York, United States; e Zhejiang University, MOE Frontier Science Center for Brain Research and Brain Machine Integration, Hangzhou, China; f Zhejiang University, NHC and CAMS Key Laboratory of Medical Neurobiology, Hangzhou, China

**Keywords:** infrared neural stimulation, cell subtypes, calcium imaging, population response, neurostimulation

## Abstract

**Significance:**

Pulsed infrared neural stimulation (INS, 1875 nm) is an emerging neurostimulation technology that delivers focal pulsed heat to activate functionally specific mesoscale networks and holds promise for clinical application. However, little is known about its effect on excitatory and inhibitory cell types in cerebral cortex.

**Aim:**

Estimates of summed population neuronal response time courses provide a potential basis for neural and hemodynamic signals described in other studies.

**Approach:**

Using two-photon calcium imaging in mouse somatosensory cortex, we have examined the effect of INS pulse train application on hSyn neurons and mDlx neurons tagged with GCaMP6s.

**Results:**

We find that, in anesthetized mice, each INS pulse train reliably induces robust response in hSyn neurons exhibiting positive going responses. Surprisingly, mDlx neurons exhibit negative going responses. Quantification using the index of correlation illustrates responses are reproducible, intensity-dependent, and focal. Also, a contralateral activation is observed when INS applied.

**Conclusions:**

In sum, the population of neurons stimulated by INS includes both hSyn and mDlx neurons; within a range of stimulation intensities, this leads to overall excitation in the stimulated population, leading to the previously observed activations at distant post-synaptic sites.

## 1 Introduction

Understanding complex neural circuits and their relationship to specific behaviors involves precise temporal and spatial modulation of neuronal subtypes. Non-genetic near-infrared optical stimulation was one of the promising non-invasive neural interface technologies for the brain.^1^^–^^5^ Recently, pulsed infrared neural stimulation (INS) technique has been introduced as a method capable of modulating neural activity safely and reversibly.^1^ In contrast to effects induced by alternative wavelengths of infrared stimulation (e.g., 808 nm,^2^ 980 nm,^3^ 5.6  μm^4^^,^^5^), the pulsed delivery of ∼1.875  μm infrared wavelengths leads to a focal delivery of heat and rapid absorption by water.^6^ When delivered via 200  μm fiber optic in short pulse trains (0.25 ms, 200 Hz, 0.5 s), this highly focal (submillimeter) optical approach has provided a unique way of functional column-specific stimulation in primate cortex.^7^ Thus, the advantages of INS over conventional electrical stimulation include high spatial selectivity, contactless delivery, and importantly for primate and human application, neuromodulation of brain sites without prior opsin expression.^8^^,^^9^ Furthermore, with this precision of targeted optical fiber stimulation and MRI compatibility, focal INS combined with MRI can be used for *in vivo* mapping of brain networks in primates^10^^–^^12^ and holds promise for neuromodulation in awake behaving monkeys. While such applications have demonstrated great promise for circuit neuromodulation *in vivo*, its mechanism of action or effect on individual cell types is currently still poorly understood.

There is now a growing body of evidence showing that INS leads to neuromodulation. INS has been shown to induce excitatory and inhibitory neuronal responses in anesthetized rodents, as assessed with electrophysiology, intrinsic signal optical imaging, and calcium imaging *in vivo*.^13^^,^^14^ INS on visual cortex in anesthetized macaque monkeys produced responses typical of visually induced cortical intrinsic signals^7^ and furthermore led to selective modulation of functionally matched ocular dominance domains, consistent with activation of local cortico-cortical connections. The demonstration that INS in ultrahigh-field MRI could lead to activation of anatomically predicted mesoscale global brain sites in the macaque monkey further suggested that projection cells (excitatory pyramidal neurons) are being activated by INS.^10^^–^^12^ These INS-induced responses have been shown to be intensity- and duration-dependent.

Despite this compelling evidence, it has been challenging to directly demonstrate neuronal response electrophysiologically. One issue, referred as the Becquerel effect, is that the direct heating of the recording electrode contaminates the neuronal response with heat-induced current in the electrode. Cayce et al. used calcium imaging *in vivo* with simultaneous INS in anesthetized rodents and observed intracellular calcium signals in cortical astrocytes and apical dendrites on the brain surface.^14^ Kaszas et al. used two-photon calcium imaging with the genetically encoded calcium indicator Syn-GCaMP6f and showed that INS induces weak intracellular calcium signals in neurons in anesthetized mouse cortex *in vivo*.^15^ To date, our understanding of neuronal response is still rudimentary. The underlying mechanism of action is unknown^16^^–^^23^ and the effect on responses of different neuronal subtypes as well as different physiological states *in vivo* at a cellular level is still lacking. In particular, even though INS has been shown to induce BOLD activation at distant cortical sites in fMRI studies, little is known about the cellular circuits contributions to this functional connectivity result.

To address how INS affects single neurons *in vivo* and to examine effects on different cell subtypes, we conducted two-photon imaging of neuronal calcium response to INS at single-cell resolution in layers 2/3 of mouse somatosensory cortex. Calcium response of hSyn- and mDlx-labeled neuronal subtypes was examined using specific genetically encoded calcium indicators GCaMP6s. We found that INS induced robust, intensity-dependent modulation of neuronal calcium reflectance change which occurred precisely in phase with the frequency of pulse train repetitions. In anesthetized mouse, hSyn neurons exhibited positively deflecting responses to INS. Surprisingly, mDlx neuron population contained varied responses, some of which exhibited negative going response, and may reflect diversity in the inhibitory neuron population. Thus, these data establish the effectiveness of INS on both hSyn and mDlx neurons and possible dependence on cell subtypes. The implications of this finding are discussed.

## 2 Materials and Methods

### 2.1 Animals

All experimental animals were approved by the Zhejiang University Animal Experimentation Committee and were completed in compliance with the National Institutes of Health Guide for the Care and Use of Laboratory Animals. Female C57BL/6J mice (8 to 10 weeks old) were group-housed (3 per cage) on a 12 h light-dark cycle and provided with food and water *ad libitum*.

### 2.2 Virus Injection

For hSyn neurons imaging, we injected 200 nL rAVV2/9-hSyn-GCaMP6s-WPRE-hGH-pA ($\text{titer}=1.03\times 10^{13}\;\mathrm{vg}/\mathrm{ml}$, PT-0145, BrainVTA) per site; for mDlx neurons imaging, we injected 200 nL rAAV2/9-mDlx-GCaMP6s-WPRE-hGH-pA ($\text{titer}=5.48\times 10^{12}\;\mathrm{vg}/\mathrm{ml}$, PT-2757, BrainVTA) per site. For co-localization experiment, we injected 200 nL rAAV2/9-mDlx-GCaMP6s-WPRE-hGH-pA ($\text{titer}=5.48\times 10^{12}\;\mathrm{vg}/\mathrm{ml}$, PT-2757, BrainVTA) and 200 nL rAAV2/9-hSyn-NES-jRGECO1a-WPRE-hGH-pA ($\text{titer}=5.82\times 10^{12}\;\mathrm{vg}/\mathrm{ml}$, PT-1593, BrainVTA), respectively.

### 2.3 Cranial Window

Mice were anesthetized with isoflurane (5% inhalation, mixed with fresh air, 0.5  L/min) and placed in a stereotactic frame, after which isoflurane was maintained at 2% throughout the surgical procedure. The body temperature was maintained at 37°C by a heating pad during the procedure. A craniotomy (measuring 4 mm in diameter, around the coordinate of 0.3 mm posterior and 2.3 mm lateral from bregma) was performed exposing the right somatosensory cortex using a dental drill. The dura was carefully removed, and artificial cerebrospinal fluid (ACSF) was used to keep the brain tissue clean and viable. Then, we injected 200 nL of virus per site around the craniotomy center at 400  μm below the pia. About three to five sites of viruses were manually injected by a patch pipette (tip diameter of ∼15 to 20  μm). A cover glass (measuring 6 mm in diameter) was used to cover the brain and secured with medical glue at its edge. A custom-made head-plate with screw holes was glued to the skull, and the gap between the head-plate and the skull was secured with dental acrylic. A chronic imaging window was completed which can be used repeatedly immobilized in the same position in the two-photon microscopy. Mice received an intraperitoneal injection of ceftriaxone (2.5  mg/kg) and a subcutaneous injection of buprenorphine (0.05  mg/kg) for up to 3 days post-surgery and were allowed to recover for at least 3 weeks before they were imaged.

### 2.4 Two-Photon Imaging

Mice were imaged using customized two-photon microscopy (2P PLUS, Bruker Corporation) coupled with a femtosecond mode-locked Ti: Sapphire laser (80 MHz, 140 fs, Chameleon Ultra II, Coherent Inc.) with 920 nm (for GCaMP6s only) or 965 nm (for GCaMP6s and jRGECO1a). A Pockels Cell (EO-PC, Thorlabs Corporation) was used to regulate laser power. For neuron imaging, power after the 4× objective (N4X-PF, 0.13 NA, Nikon) and 16× objective (N16XLWD-PF, 0.8 NA, Nikon) was limited to a maximum of 40 mW, dependent on depth. Emission light was filtered using a bandpass 525/70 filter for GCaMP6s and a bandpass 595/50 filter for jRGECO1a, detected by two GaAsP PMTs (model H10770, Hamamatsu Photonics). Images were collected at ∼30  Hz using a resonant-galvo scanner with 512×512  pixel resolution for functional imaging. Images were collected using a galvo-galvo scanner with 1024×1024  pixel resolution for structure imaging. For two-photon imaging in anesthetized experiments, isoflurane (0.6%, mixed with fresh air, 0.5  L/min) was used to anesthetize mice during imaging. For two-photon imaging in awake experiments, 3 days of routine handling of the mice to acclimate them to the imaging system and immobilization device, which greatly helped to reduce animal motion.

### 2.5 Infrared Neural Stimulation

For all INS experiments, a single wavelength laser (FC-W series, CNI) coupled with multimode fiber (MM200, Newdoon; 200  μm core diameter, 0.37 NA) was used to provide an 1875 nm infrared beam. The laser fiber was placed into a custom-built micromanipulator and under two-photon microscopy. Effective laser pulses (wavelength, 1875 nm; peak radiant exposure, from 0.1 to $1\;J/{\mathrm{cm}}^{2}$ per pulse at fiber tip) were calibrated with a power meter (PM320E, Thorlabs Corporation; coupling with thermal power sensor, S401C, Thorlabs Corporation) before every experiment and transmitted through an optical fiber positioned at a ∼45  deg angle to the brain surface. For multiple pulse train stimulation, each pulse train contains six bursts of 0.5 s duration (0.25 ms pulse width per pulse at 200 Hz, 100 pulses) and 2.5 s interval presented once every 60 s. In a single trial, the first 10 s was the baseline, following 18 s INS application and 32 s convalescence time. The laser powers were presented in randomized order to prevent trends. For long-lasting pulse train stimulation, the pulse train contains 2 s duration (0.25 ms pulse width per pulse at 200 Hz, 400 pulses) once every 30 s. In a single trial, the first 3 s was the baseline, following 2 s INS application and 25 s convalescence time. The two-photon system was used to initiate a transistor-transistor logic (TTL) signal, sending it to the laser to synchronize the infrared neural modulation and imaging.

### 2.6 Monte Carlo Simulation for Infrared Light

To simulate light propagation in planar multi-layered tissues, we modeled tissues in a cube of 4 mm slide length. A 4×4×4  mm cube of tissues in Cartesian coordinates was generated, discretizing space in 10  μm steps, which we modified the codes for our infrared light (λ=1875  nm) propagation.^24^^,^^25^ The infrared light propagation emitted from the optical fiber (200  μm core diameter, 0.37 NA) and recording of simulated quantities were performed through three-layered tissue structures (the first layer is ACSF; the second layer is glass; the third layer is cortex) with a Cartesian-grid voxel-based method, which allows assignment of optical properties, including absorption coefficient ($\mu_{a}$ $\left[{\mathrm{mm}}^{-1}\right]$), scattering coefficient ($\mu_{s}$ $\left[{\mathrm{mm}}^{-1}\right]$), and anisotropy of scattering (g [dimensionless]) to each individual voxel. The current simulation code was fully implemented with MATLAB (version R2020b, MathWorks).

### 2.7 Data Analysis

Acquired time-lapse two-photon images were read and analyzed in FIJI (Fiji Is Just ImageJ, NIH), Cellpose (version 2.0), and MATLAB (version R2020b, MathWorks). Movement artifacts in the xy plane were corrected by using the template_matching plugin in FIJI.^26^^,^^27^ Cellpose was used to detect neuron soma except for neuropil and perform cellular segmentation to get masks.^28^ FIJI registers raw movies, uses masks, and extracts calcium fluorescence traces. Fluorescence changes (F) were calculated by averaging the corresponding pixel values in each specified ROI mask. In the following analysis, we used our self-written MATLAB codes. Relative fluorescence changes $\Delta F/F_{0}=(F-F_{0})/F_{0}$ were calculated as calcium transient, where the baseline fluorescence $F_{0}$ was estimated as the first several seconds before stimulation of the entire fluorescence recording. For typical example in Figs. 2(a), 3(d), 3(e), 5(a), and 6(a), Kalman filter^29^ was used to smooth the data in MATLAB.

For multiple pulse train stimulation, two special indices were used to evaluate the averaged response caused by the INS for each cell. First, the correlation value was defined as the correlation between the typical response curve (the averaged INS response at $0.76\;J/{\mathrm{cm}}^{2}$) and every trial-averaged response curve for a single cell across all intensities during the INS [as shown in Fig. S4(a) in the Supplementary Material]. A positive calcium response (excitatory effect by INS) was defined by the correlation value >0.2. A negative calcium response (inhibitory effect by INS) was defined by the correlation value <−0.2 [threshold shown in Fig. S4(b) in the Supplementary Material]. Second, the averaged peak-to-peak amplitude of calcium transient ($\Delta F/F_{0}$) was used as an index of neuronal response during the INS. We calculated six peaks during six pulse trains (p1,p2,p3,p4,p5,p6) and averaged these six peak values as the amplitude value [as shown in Fig. S4(c) in the Supplementary Material]. When the correlation value was positive, its amplitude was positive which means an increase induced by INS. When the correlation value was negative, its amplitude was negative which means a decrease induced by INS. For long-lasting pulse train stimulation, the total peak area under the curve during the 3 to 10 s period was measured as a stimulated effect analyzed by prism [Fig. 4(f) for calculation example]. This value is affected by several parameters in the analysis dialog in prism: the definition of baseline (baseline = 0) and the definition of peaks too small to count (ignore peaks that are less than 10% of the distance from minimum and maximum), which are the default parameters in prism.

### 2.8 Statistical Analysis

All data are presented as means ± SEM. A paired Wilcoxon test was performed to estimate the statistical differences in Fig. 4(f) and Fig. S3 in the Supplementary Material. An unpaired Mann–Whitney test was performed to estimate the statistical differences in Fig. S2 in the Supplementary Material. The comparison was analyzed using the Wilcoxon test when they can be paired one by one; otherwise, a Mann–Whitney test was used as indicated. In all tests, p<0.05 is considered statistically significant and unmarked represents no significance. All statistical analyses were performed in prism (version 8.0, GraphPad Software). No additional methods were used to determine whether the data met the assumptions of the statistical approach. The statistical details of each experiment can be found in the figure legends and tables.

## 3 Results

We measured the INS-evoked *in vivo* calcium activity of populations comprising hSyn versus mDlx neuronal subtypes expressing the hSyn-GCaMP6s and mDlx-GCaMP6s, respectively,^30^ as well as a two-color multi-labeling approach. We applied a robust version of a common pulsed INS paradigm (pulse trains consisting of short pulses 0.25 ms, high frequency 200 Hz, short pulse train duration 0.2 to 0.5 s). Stimulation was applied in sets of six pulse trains to assess reliability of response (Fig. S1 in the Supplementary Material showed correction of the applied laser energy levels for the glass).

### 3.1 Viewing Calcium Activity of Two Types of Neurons in Mouse Cortex *In Vivo*

**Fig. 1:**
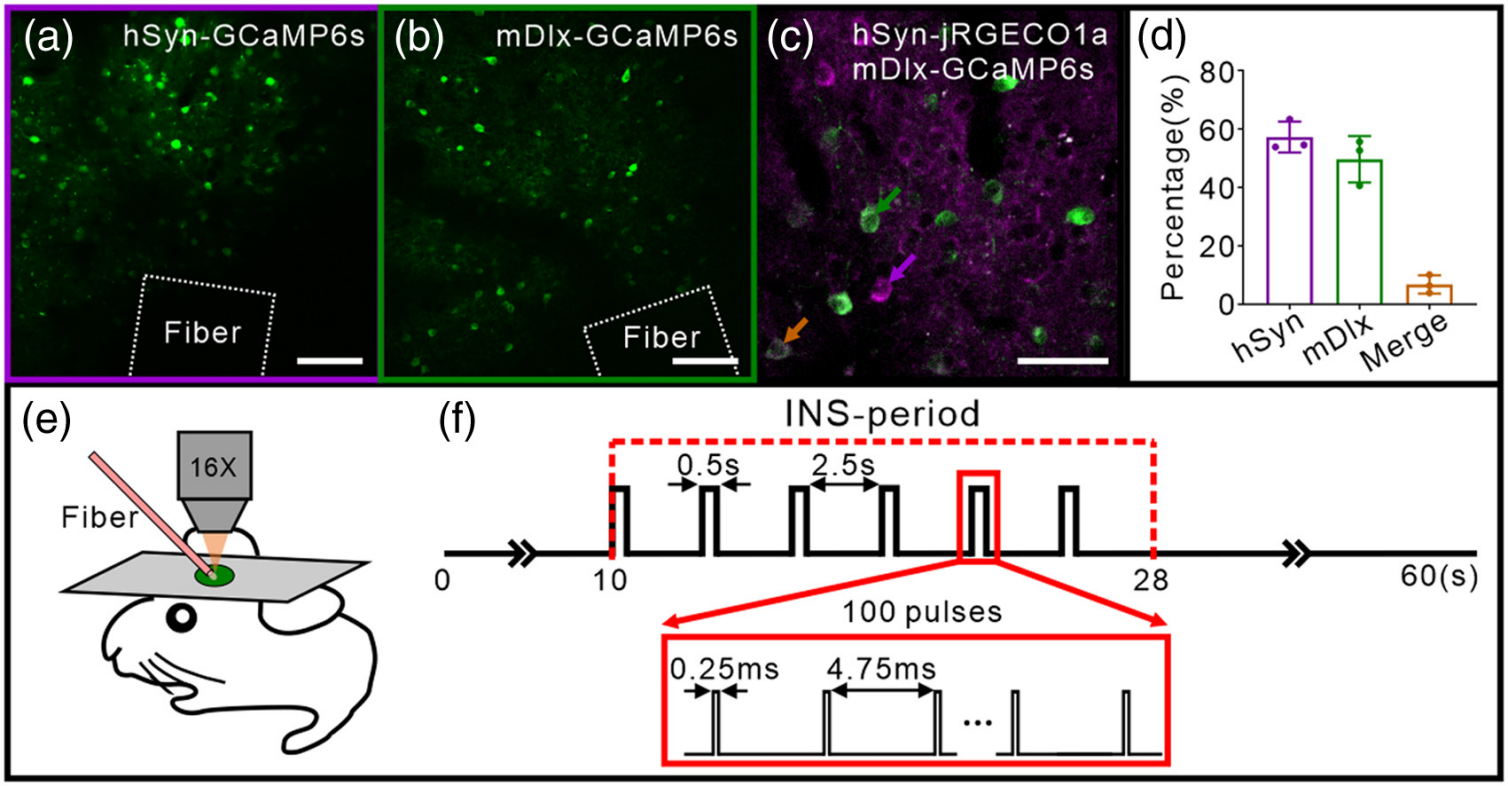
INS-TPM setup and specific subtypes calcium imaging of INS-induced neuronal response in mouse cortex. (a) hSyn-GCaMP6s expression in mouse somatosensory cortex labels hSyn neurons. The white dotted box indicates INS fiber tip position. Scale bar, 100  μm. (b) mDlx-GCaMP6s expression in mouse somatosensory cortex labels mDlx neurons. The white dotted box indicates INS fiber tip position. Scale bar, 100  μm. (c) hSyn-jRGECO1a (purple) and mDlx-GCaMP6s (green) co-expression in the same mouse somatosensory cortex labels different population neurons, respectively. Scale bar: 50  μm. (d) Proportion of hSyn-jRGECO1a (purple) and mDlx-GCaMP6s (green) expression neurons, respectively. Error bars are mean ± SEM. (e) INS-TPM setup, the mouse brain was fixed under the TPM and the somatosensory cortex was covered by a glass. The optical fiber (pink bar) touched on the cover glass for stimulation. (f) INS paradigm. One INS comprises six pulse trains and each train comprises 100 pulses at single energy. Train duration: 0.5 s, train interval: 2.5 s, repeat six times; pulse width: 0.25 ms, repetition rate: 200 Hz; radiant exposure: 0.16 to $0.76\;J/{\mathrm{cm}}^{2}$.

For our initial attempts to investigate the properties of pan-neuronal calcium activity to INS in mouse somatosensory cortex, we employed the viral labeling strategies that AAV-expressed GCaMP6s^30^^,^^31^ under the pan-neuronal promoter human synapsin (hSyn) packaged in serotype 2/9 (AAV2/9-hSyn-GCaMP6s), which mainly produces robust expression of GCaMP in excitatory neurons^30^^,^^32^ [Fig. 1(a)]. To further observe the specific inhibitory neurons responding to INS, we adopted AAV-expressed GCaMP6s under an enhancer (mDlx) that induces expression in GABAergic neurons across a wide range of species, including mice^30^^,^^32^^,^^33^ [Fig. 1(b)]. To examine whether these populations are distinct, we co-labeled the two cell subtypes using hSyn-jRGECO1a and mDlx-GCaMP6s in the same field of view (FOV) in the same mouse cortex [Fig. 1(c); Fig. S2 in the Supplementary Material showed spontaneous activity variations in two subtypes]. This revealed very few co-labeled neurons [Fig. 1(d), 6.8±1.8%; total 383 neurons in n=3 FOVs from two animals; 219 hSyn-labeled neurons, 189 mDlx-labeled neurons, and 25 merged neurons], indicating the cell populations labeled in Figs. 1(a) and 1(b) were distinct populations.

**Fig. 2:**
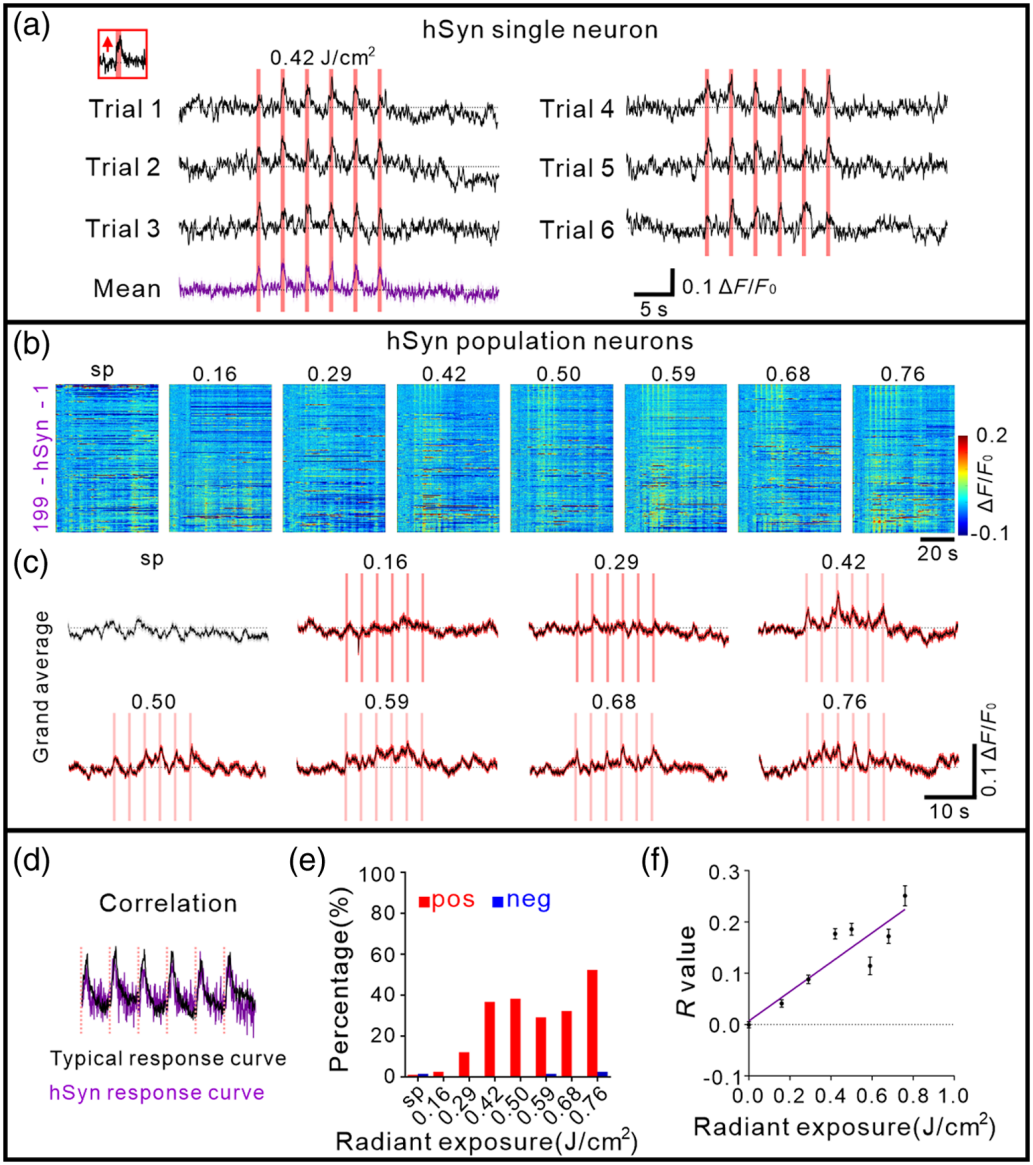
INS-induced calcium activity of hSyn neurons in anesthetized mice. (a) Positive response of a typical hSyn neuron to INS (radiant exposure: $0.42\;J/{\mathrm{cm}}^{2}$). The pink bar indicates the INS period. The inset panel indicated the enlarged time response during the first INS pulse train. Bottom purple curve was mean. (b) All trial-averaged responses of hSyn neurons (n=199 neurons in three anesthetized mice) and a pseudo-colored plot summarizing the trial-integrated calcium activity trace for each neuron during INS intensity at 0.16, 0.29, 0.42, 0.50, 0.59, 0.68, and $0.76\;J/{\mathrm{cm}}^{2}$, sorted by correlation during the INS period, respectively. (c) A grand average of spontaneous and INS-induced calcium activity traces for all neurons corresponding to b. (d) Percentage of positive calcium activity induced by INS in hSyn neurons (red, 1.0%, 2.5%, 12.1%, 36.7%, 38.2%, 29.1%, 32.2%, and 52.3% at 0, 0.16, 0.29, 0.42, 0.50, 0.59, 0.68, and $0.76\;J/{\mathrm{cm}}^{2}$, respectively). pos, positive response; neg, negative response. (e) Calculation of correlation index for hSyn neurons. (f) Correlation of calcium responses curve in hSyn illuminated-region neurons (fitting curve, Y=0.29×X+0.01, $R^{2}=0.81$, p=0.0024). Data represent mean ± SEM in Table S1 in the Supplementary Material.

**Fig. 3:**
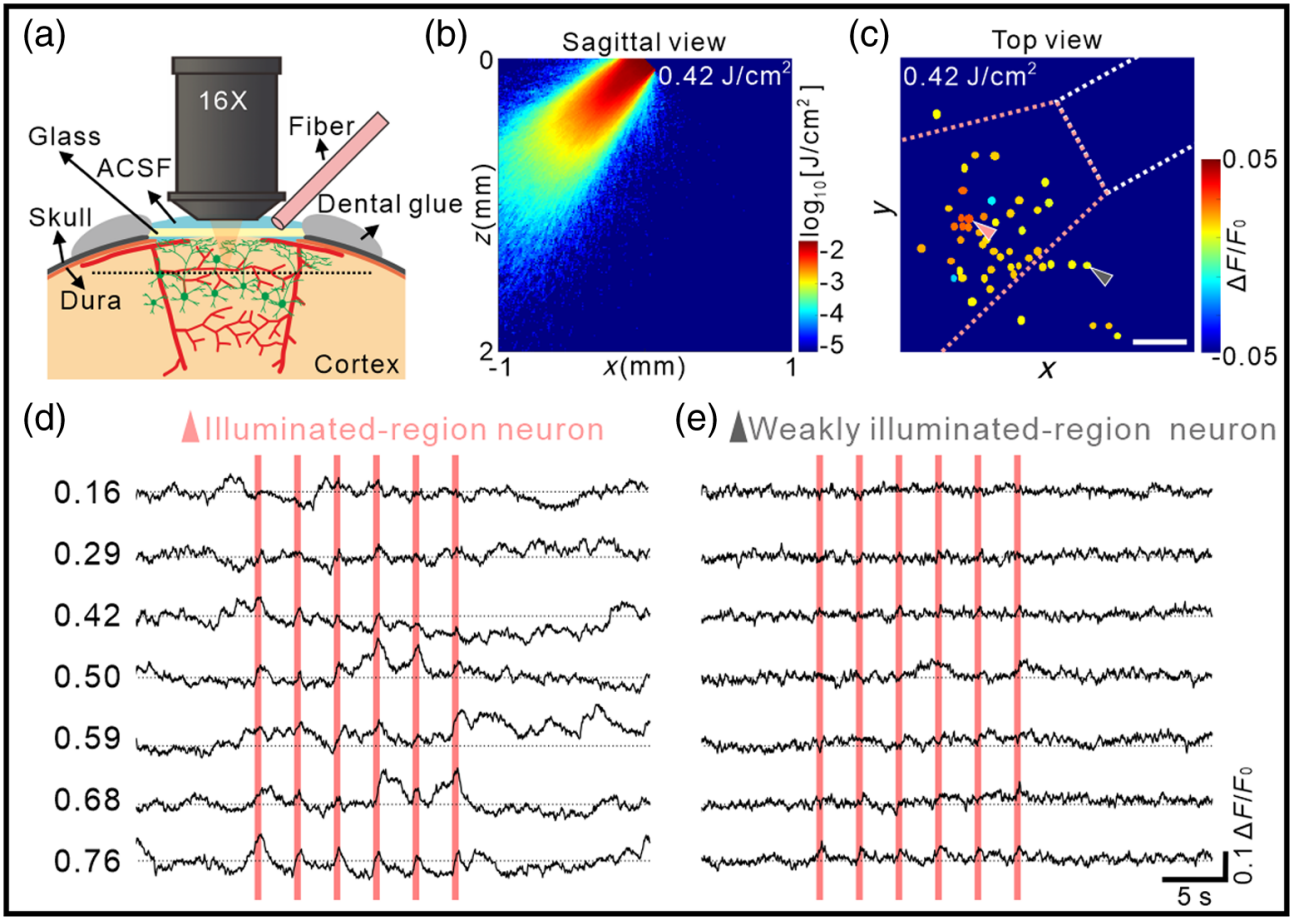
Focal effect of calcium responses to INS in anesthetized mice. (a) Illustration of the optical fiber placement and the imaging of calcium transients. An optical fiber tilted at an angle of ∼45  deg to the horizontal plane delivers infrared light to the cortex through the cover glass. (b) Theoretical infrared light distribution from a 200  μm core diameter fiber based on a Monte Carlo simulation. (c) Color coding of hSyn neurons spatial distribution map based on amplitude induced by INS. The white dotted box indicates the fiber position. The pink dotted conoid indicates the illuminated region. The red box indicates the FOV in two-photon imaging (illuminated-region neuron: n=40, pink triangle indicates the neuron in light direct exposure areas; weakly illuminated-region neuron: n=9, gray triangle indicates the neuron outside illuminated regions). Scale bar, 100  μm. (d), (e) A representative example of INS-induced calcium transient of an illuminated- (d, pink triangle in c) and weakly illuminated-region (e, gray triangle in c) neuron over different laser radiant exposures, respectively. The pink bar indicates the INS period. Data (n=6 trials) represent the mean.

**Fig. 4:**
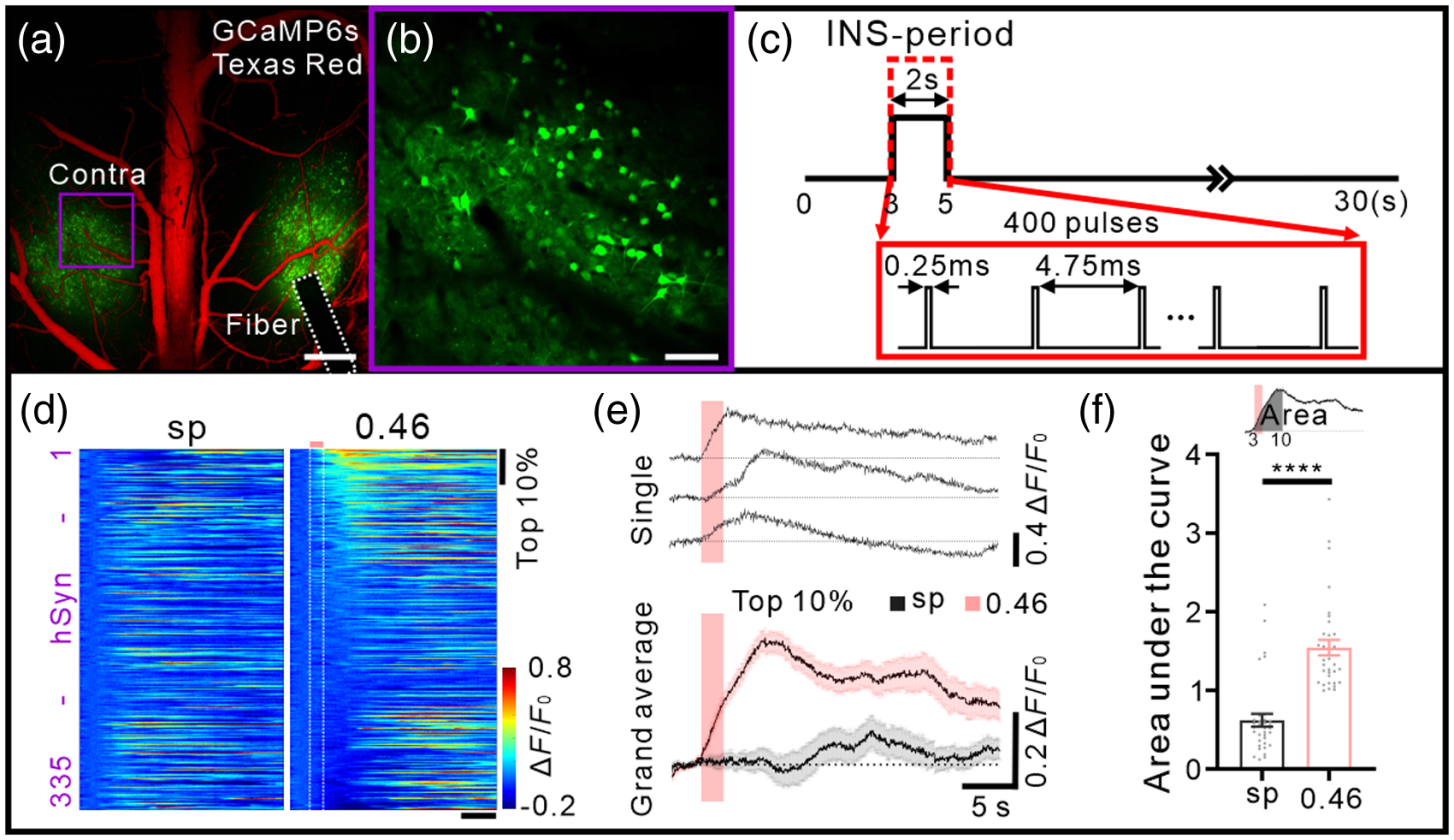
INS-induced calcium activity in the contralateral cortex in awake mice. (a) Setup of contralateral imaging during INS. The white dotted box indicates INS fiber. The purple box indicates contralateral FOV. Scale bar, 500  μm. (b) hSyn-GCaMP6s expression in mouse contralateral cortex in awake mice. Scale bar, 50  μm. (c) INS paradigm. One INS comprises a long-lasting pulse train (400 pulses). Train duration: 2 s; pulse width: 0.25 ms, repetition rate: 200 Hz; radiant exposure: $0.46\;J/{\mathrm{cm}}^{2}$. (d) All trial-averaged responses of hSyn neurons in the contralateral cortex (n=335 neurons in three awake mice) and a pseudo-colored plot summarizing the trial-integrated calcium activity trace for each neuron during spontaneous baseline and INS, sorted by area under the curve during the 3 to 10 s period. (e) An example of INS-induced calcium activity traces and a grand average of top 10% neurons (n=34) corresponding to (d). (f) Area of calcium responses curve (during the 3 to 10 s period) in top 10% neurons between spontaneous baseline and INS-induced activity (area, sp: 0.62±0.08, $0.46\;J/{\mathrm{cm}}^{2}$: 1.54±0.10, p<0.0001). Wilcoxon test; data represent mean ± SEM.

**Fig. 5:**
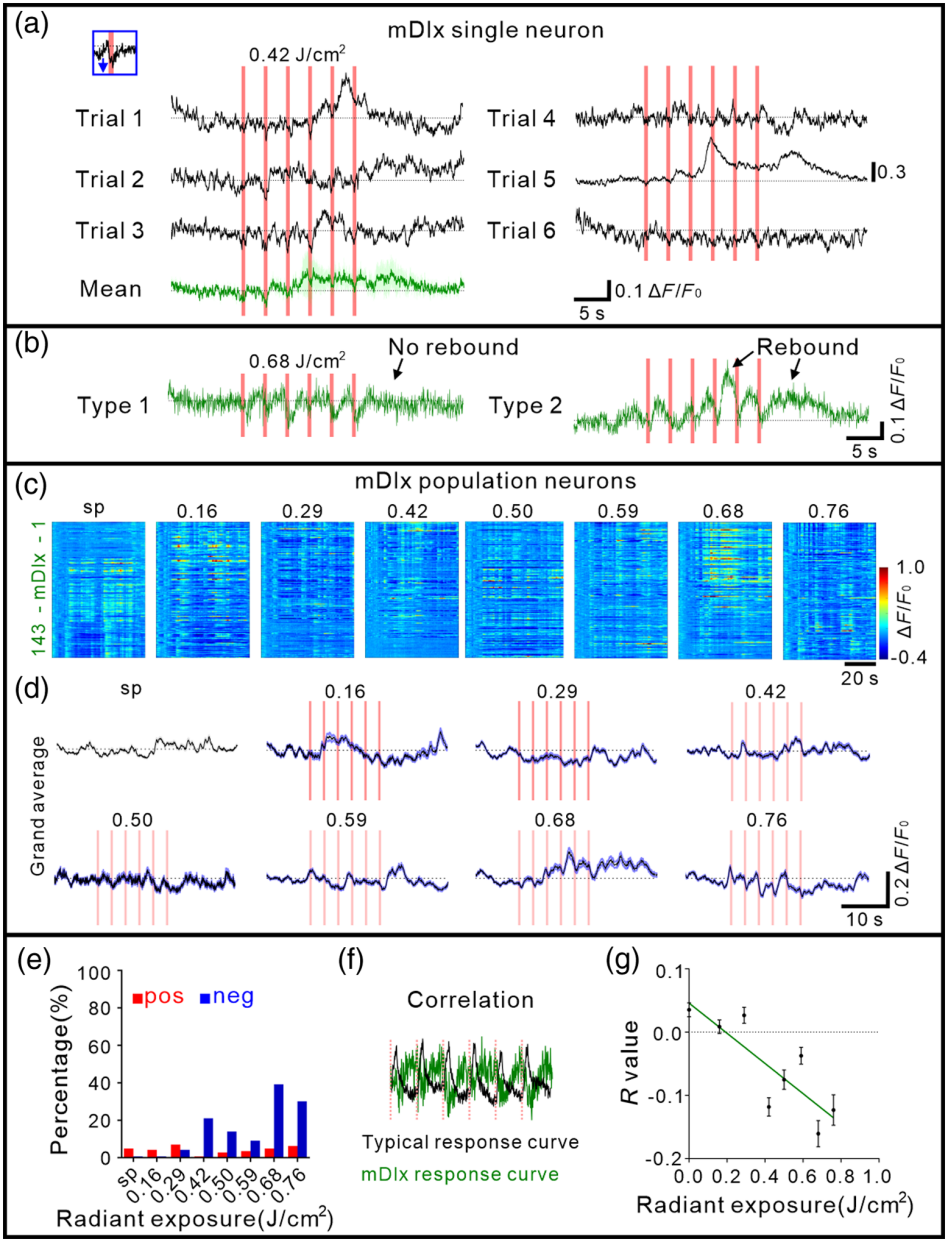
INS-induced calcium activity of mDlx neurons in anesthetized mice. (a) Negative response of a typical mDlx neuron to INS (radiant exposure: $0.42\;J/{\mathrm{cm}}^{2}$). The pink bar indicates the INS period. The inset panel indicated the enlarged time response during the first INS pulse train. Bottom green curves were mean. (b) Two types of negative responses of mDlx neurons. (c) All trial-averaged responses of mDlx neurons (n=143 neurons in three anesthetized mice) and a pseudo-colored plot summarizing the trial-integrated calcium activity trace for each neuron during INS intensity at 0.16, 0.29, 0.42, 0.50, 0.59, 0.68, and $0.76\;J/{\mathrm{cm}}^{2}$, sorted by correlation during the INS period, respectively. (d) A grand average of spontaneous (gray) and INS-induced calcium activity traces for all neurons corresponding to (b). (e) Percentage of negative calcium activity induced by INS in mDlx neurons (blue, 0.7%, 0.7%, 4.2%, 21.0%, 14.0%, 9.1%, 39.2%, and 30.1% at 0, 0.16, 0.29, 0.42, 0.50, 0.59, 0.68, and $0.76\;J/{\mathrm{cm}}^{2}$). pos, positive response; neg, negative response. (f) Calculation of correlation index for mDlx neurons. (g) Correlation of calcium responses curve in mDlx illuminated-region neurons (fitting curve, Y=−0.24×X+0.05, $R^{2}=0.69$, p=0.0104). Data represent mean ± SEM in Table S1 in the Supplementary Material.

We employed two-photon calcium imaging to visualize these two subtypes of neuronal calcium activity during INS application separately. In these experiments, we applied a variant of previously established stimulation parameters that were shown to induce neuromodulation effectively.^13^^,^^14^^,^^34^ In our previous studies,^7^ using a 200-μm optical fiber aimed at targeting 200 to 300  μm sized cortical columns, we used trains of very brief pulses (short pulse 0.25 ms, high frequency 200 Hz, short pulse train duration 0.5 s, radiant exposure $0.16-0.76\;J/{\mathrm{cm}}^{2}$ per pulse, and ISI of 2.5 s); this delivered small boluses of heat absorption (thermal confinement regime) to the tissue. Here, to better observe the reliability of response to INS in mouse [Fig. 1(e)], we modified the previous paradigm of three to four pulse trains^11^ to six such pulse trains and recorded for 60 s per trial [Fig. 1(f)]. Neurons maintained normal activity after INS application with effective radiant energy levels (cf., Fig. S3 in the Supplementary Material). Below, we present results from hSyn neurons (Figs. 2–4) and then from mDlx neurons (Fig. 5).

### 3.2 INS Induced Calcium Activity: hSyn Neurons

Here, we exhibited the effects of INS on hSyn neurons in anesthetized mice. As shown in Fig. 2(a) with a single hSyn neuron, using real-time (∼30  Hz acquisition rate) two-photon imaging over the entire course of INS [1 trial total 60 s: 10 s baseline, 18 s INS period, 32 s recovery period, see Fig. 1(f)], we found that indeed the pulse trains (each indicated by pink vertical line) modulated neuronal calcium response (black traces) in phase with the pulse train delivery. Similar to a previous study,^15^ we observed positive deflections from baseline in the calcium signal responses of hSyn neurons [Fig. 2(a), red arrow in top left inset]. As shown in the six trials, which is clearly seen in the mean of six such trials [bottom purple trace in Fig. 2(a)], this was a consistent response across the six successive pulse trains in each trial and consistent across trials.

*In vivo* calcium responses were recorded in 199 hSyn neurons in anesthetized mice [n=3 mice, Fig. 2(b)]. Previous studies characterizing INS have indicated that radiant exposure is a primary parameter for determining the strength of response.^7^^,^^10^^,^^13^^,^^15^ We delivered different laser radiant exposures of INS; these values (0.16 to $0.76\;J/{\mathrm{cm}}^{2}$) were guided by irradiance values determined to be effective in our previous studies.^10^^,^^11^^,^^13^^,^^14^ INS intensities were applied at radiant exposures of 0.16, 0.29, 0.42, 0.50, 0.59, 0.68, and $0.76\;J/{\mathrm{cm}}^{2}$ (INS intensity correction of glass shown in Fig. S1 in the Supplementary Material). Populations of hSyn neurons responded robustly to INS in anesthetized state [mean traces shown in Fig. 2(c)] and most of them exhibited the positive going behavior [n=5, 24, 73, 76, 58, 64, and 104 out of 199 neurons at seven intensities, respectively; percentage shown in Fig. 2(d)]. Thus, under anesthesia, hSyn neurons predominantly respond with reliable and reproducible positive deflections.

To understand the different aspects of neuronal behavior to INS, we quantified characteristic correlation between the standard response (averaged positive responses to INS at $0.76\;J/{\mathrm{cm}}^{2}$) and each response time course [Fig. 2(e) and see calculation paradigm in Fig. S4(a) in the Supplementary Material for the example; for more details in Sec. 2]. For hSyn illuminated-region neurons (n=140 neurons from three anesthetized mice. The definition is described in Fig. 3; for weakly illuminated-region neurons, see Fig. S6 in the Supplementary Material), as shown in Fig. 2(f), correlation values exhibited, at the lower radiant exposure, a moderate correlation, while strong correlations emerged at higher energies. The fitted correlation curve showed an increased correlation value with the increase in laser radiant exposures (linear fitting), illustrating clear intensity dependence in Fig. 2(f). Thus, by correlation measures the effect of increasing INS intensities, for hSyn neurons, led to an increased calcium-positive response. This characterizes the intensity dependence and supports the reliability and reproducibility of INS-induced response in hSyn neurons.

### 3.3 Focal Effect of INS-Induced Calcium Activity

To examine the spatial extent of INS, we conducted Monte Carlo simulation of the INS light distribution and examined neuronal responses inside and outside this region of INS illumination. These analyses were conducted based on our experimental setup [cartoon diagram shown in Fig. 3(a)]. Monte Carlo simulation was performed based on illumination with a 45 deg angle fiber to estimate the theoretical infrared light distribution [Fig. 3(b), for detail see Fig. S5 in the Supplementary Material]. In Fig. 3(c), the fiber position (shown as a white dotted box) is shown in relation to all neurons in the FOV of two-photon imaging. Based on the distance of the neuron from the fiber tip center measurements (three-dimensional space distance), the relationship between amplitude and distance is hard to quantify. So, we next defined the light region based on Monte Carlo simulated light distribution (pink dotted trapezoid) and binned these neuronal responses into “illuminated-region” versus “weakly illuminated-region.” After color coding each neuron by amplitude [see color scale in Fig. 3(c) and calculation paradigm in Fig. S4(c) in the Supplementary Material for the example], we found a focal effect across the population in anesthetized mice. Neurons within the illuminated region are categorized as “illuminated-region” neurons [an example of hSyn neuron in Fig. 3(d), the position shown with pink triangle in Fig. 3(c)] and out of the illuminated region as “weakly illuminated-region” neurons [an example of hSyn neuron in Fig. 3(e), the position shown with gray triangle in Fig. 3(c)]. The illuminated-region neuron exhibits strong upward deflections (positive responses). The weakly illuminated-region neuron, in contrast, has a relatively weak response across all intensities tested. This suggests possible light intensity-related focal effects. We further examined the calcium response to INS in contralateral cortex.

### 3.4 INS Induced Calcium Activity in the Contralateral Cortex

We anticipated seeing a distally connected cortex activation response in addition to the local activation induced by INS, as shown in the previous fMRI study.^10^ To address this distant response of neurons, we positioned the optical fiber as before and then observed the calcium activity in the contralateral cortex [see setup of Figs. 4(a) and 4(b)]. Furthermore, we applied a long-lasting version of a common pulsed INS paradigm [400 pulses, as shown in Fig. 4(c)]. In the spontaneous state and the stimulation phase, we examined 335 hSyn neurons from three awake mice, respectively [as shown in Fig. 4(d), INS radiant exposure: $0.46\;J/{\mathrm{cm}}^{2}$]. A delayed peak is observed and lasted 5 to 10 s in the INS condition in Fig. 4(e). The activation response is shown by a quantification of the area under the curve following INS (during the 3 to 10 s period), and this allowed us to determine that the stimulation had an effect on the contralateral brain [see Fig. 4(f)]. This cellular population response can be considered to support the connection activation in the contralateral cortex of the previous INS-fMRI studies. Below we further investigate the calcium activity to INS in inhibitory neurons.

### 3.5 INS Induced Calcium Activity: mDlx Neurons

In addition to the study of hSyn neurons, we also examined responses of inhibitory (GABAergic) neurons labeled by mDlx promoter [an example of a single mDlx neuron calcium response to INS shown in Fig. 5(a)]. Interestingly, in contrast to hSyn neurons, we find that many mDlx neurons, in an anesthetized state, responded to INS with negative calcium fluorescence changes [see Figs. 5(b)–5(d), n=143 mDlx neurons from three mice]. Responses also appeared quite heterogeneous [evidenced by multiple examples of mDlx neuron responding to INS in Fig. 5(b) and the larger variability in response across neurons in Fig. 5(c), compared with Fig. 2(b)], perhaps due to diversity of GABAergic neurons.^35^ Out of 143 mDlx neurons recorded in anesthetized mice, using INS intensities at radiant exposures of 0.16, 0.29, 0.42, 0.50, 0.59, 0.68, and $0.76\;J/{\mathrm{cm}}^{2}$, up to 40% exhibited negatively going responses [Fig. 5(e)].

For mDlx illuminated-region neurons (n=88 neurons from three anesthetized mice, for weakly illuminated-region neurons, see Fig. S6 in the Supplementary Material), we quantified correlation as defined above for INS-induced calcium activity [Fig. 5(f) and see calculation paradigm in Fig. S4(a) in the Supplementary Material for the example]. The index is negative, reflecting the negative reflectance changes in mDlx neurons. The fitted curves for correlation exhibited increasing negative values with increasing radiant exposures [Fig. 5(g)]. This intensity dependence was observed for correlation. Thus, for mDlx neurons, increasing INS intensities led to increased negative calcium responses during the pulse train delivery period. This signifies that the unusual negative calcium is not only reproducible but responsive to intensity in a predictable manner. At a population level, it appears that INS produces negative calcium transients in mDlx neurons.

These results suggest that, in both hSyn and mDlx neurons, neuronal calcium response to focal INS is observed primarily within the region of INS illumination.^14^^,^^15^ This further supports that neuronal response due to INS is quite focal and intensity-dependent.

## 4 Discussion

### 4.1 Summary

Previous studies have shown that pulsed INS leads to neural response in the peripheral and central nervous system. However, the cellular basis of this response is not well understood. In this study, we examined the effect of INS on excitatory and inhibitory cortical neurons (hSyn-GCaMP6s and mDlx-GCaMP6s) using two-photon calcium imaging in mouse somatosensory cortex. Our results describe robust and reproducible calcium signals in single cells. However, responses to INS in excitatory and inhibitory neurons were very different in anesthetized mice. For hSyn neurons, our results describe robust and reproducible positive deflecting calcium signals in single cells. In contrast, for mDlx neurons, INS reliably induces a negative deflection during the light delivery. In sum, although the mechanism underlying INS effects on cellular response requires further study, our study definitively establishes that single neurons in cerebral cortex, both excitatory and inhibitory cortical neurons, are robustly and reproducibly induced by INS. This puts INS on more solid ground for applications in neuroscience, engineering, and medicine.

### 4.2 Fluoro-Thermal Effects During the INS

Historically, estimates of temperature changes induced by INS have relied on modeling and Monte Carlo simulations.^36^ We and others have also used FLIR cameras for experimental assessment and obtained changes of ∼1°C (Roe Lab, unpublished data). In *ex vivo* rat brains, MRI thermometry reveals that the temperature increase was quite confined and estimated to be below ∼1.6°C for the highest $1\;J/{\mathrm{cm}}^{2}$ intensity (Roe Lab, data not shown). The temperature rise has several auxiliary effects in addition to INS physiological effects, including fluoro-thermal effects on GCaMP6s fluorescence proteins and thermal lensing in the water. However, these effects generally result in rapid fluorescence dips.^37^^,^^38^ To address the concern that the dip is due to INS-induced thermal effect on the calcium indicator, we tested the GCaMP6s-labeled neurons in dead mice with no calcium changes. As shown in Fig. S7 in the Supplementary Material, in dead animals, neurons showed fluorescence signal decrease during the INS. These fluoro-thermal effects may overestimate the INS-induced response in mDlx neurons and underestimate the hSyn neurons in anesthetized state.

### 4.3 Comparison with Other INS Studies

Previous studies have seen both excitatory and inhibitory effects of 1875 nm INS. Electrical single-unit and multi-unit recordings in the peripheral nervous system,^39^ cochlea,^40^ and macaque and rat cerebral cortex^7^^,^^13^ have revealed the presence of excitatory effects on neurons. Patch-clamp recordings were used to explicitly characterize infrared-evoked depolarizing potentials and currents.^20^^,^^41^ Calcium imaging has also reported neuronal activation by INS, but single-neuron calcium response remained little studied.^14^^,^^15^ Using optical intrinsic signal imaging and fMRI, robust and intensity-dependent INS-induced hemodynamic signals have been recorded not only at INS sites but also at distant connected sites,^7^^,^^10^^–^^13^ a finding suggesting robust population-level pyramidal cell activation. Some studies point to inhibition evoked by pulsed INS. *In vitro* studies revealed INS increased the amplitude and frequency of spontaneous inhibitory postsynaptic currents, and that this response is mediated by GABAergic neurotransmission.^42^ Infrared inhibition has also demonstrated block of peripheral nerve conduction, suppression of axonal excitability and downstream synaptic transmission.^23^^,^^43^^–^^47^ Here, we show that INS pulse trains can induce positive deflections and negative deflections in hSyn and mDlx neurons, respectively.

### 4.4 Contribution to Population Response

**Fig. 6:**
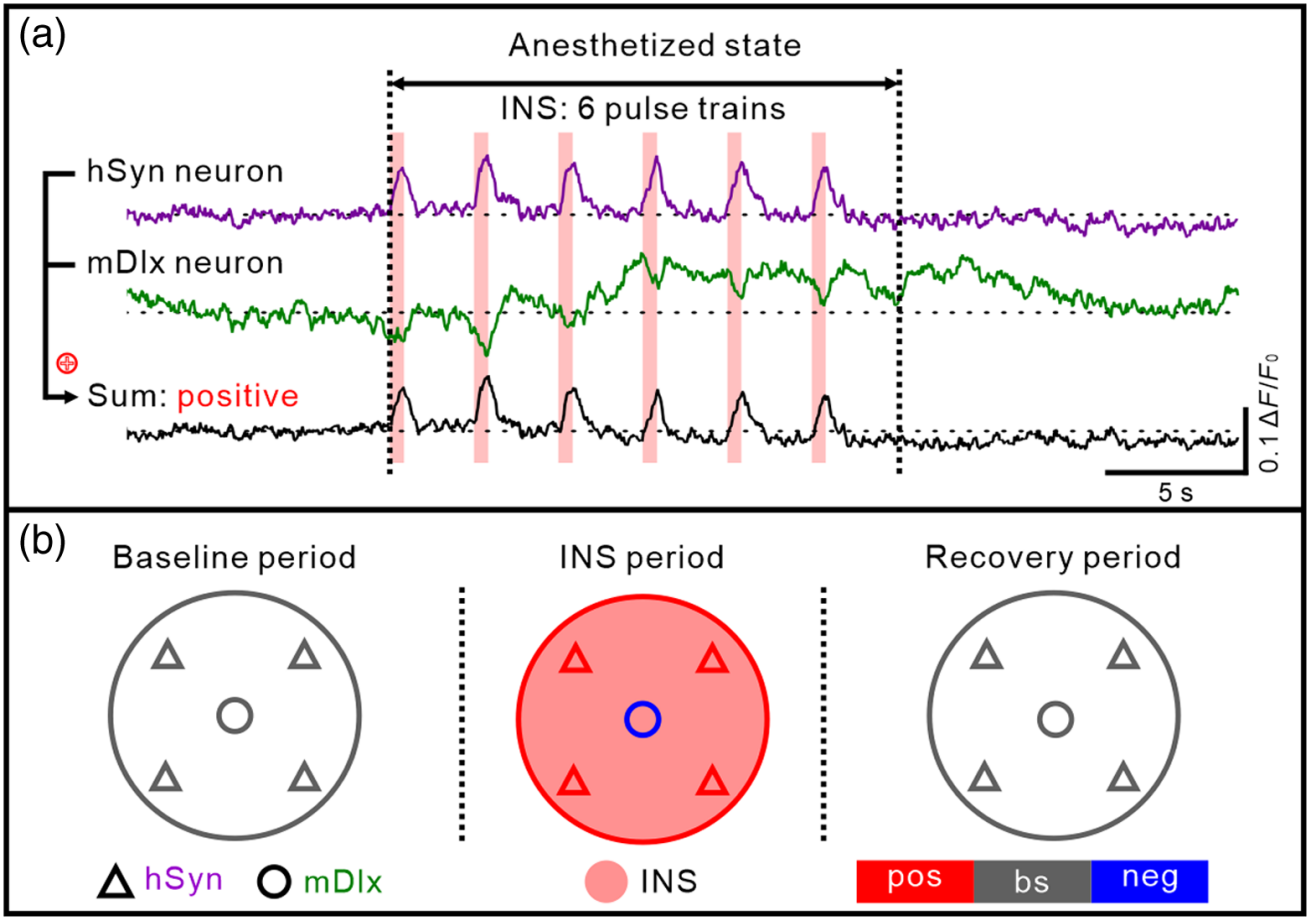
Modeling of INS-induced population neural calcium activity in anesthetized mice. (a) Time course of hSyn and mDlx neuron calcium response to INS (for example, $0.42\;J/{\mathrm{cm}}^{2}$). The sum response consists of 80% hSyn response and 20% mDlx response (purple: hSyn, green: mDlx, sum: black). The pink vertical bar indicates the INS period. (b) Population calcium response of regional hSyn neurons (triangle) and mDlx neurons (round) during different INS phases. The pink area indicates the INS irradiation region. pos, positive response (red); bs, spontaneous baseline (gray); neg, negative response (blue).

We now consider what effect these observations have at the population level in an anesthetized state. As INS is non-cell type-specific, both excitatory and inhibitory neurons within the INS optical light field are impacted. To estimate what a population response might look like, we calculated the weighted sum of our two hSyn (purple trace) and mDlx (green trace) population traces, based on the standard ratio of 80% excitatory and 20% inhibitory neurons in cerebral cortex.^48^ As shown in Fig. 6(a) (see black trace), this population time course appears quite similar to the hSyn neuron responses. Prior to INS, the population maintains a baseline spontaneous activity [proportions of cells in each phase indicated in Fig. 6(b)], and when infrared light is irradiated on excitatory (hSyn-labeled) and inhibitory (mDlx-labeled) neurons, the population is observed to oscillate in phase with the occurrence of pulse trains. This is then followed by a recovery response [Fig. 6(b), population activity model]. This population response, we suggest, is consistent with the presence of increased spiking activity during and following INS pulse train stimulation.

We further suggest that this population response is the basis for the hemodynamic responses consistently observed in INS-induced intrinsic optical imaging and BOLD fMRI studies. While the BOLD signal is a complex hemodynamic response which includes spiking and subthreshold cellular responses, as well as neuronally related and unrelated vascular contributions, the association of BOLD response with neuronal activity is generally accepted and has formed the basis for many fMRI studies. Based on a large number of intrinsic signal optical imaging studies and fMRI studies, neuronal activation leads to a brief “initial dip” (seen optically as a small and reliable 0.1% to 1% decrease in reflectance, related to the deoxygenation hemodynamic phase, often not seen in fMRI due to small size, cf. Ref. 49) and is followed by a large positive BOLD signal (related to the influx of newly oxygenated blood) with a time course that lasts several seconds before returning to baseline. INS-induced BOLD signals have been observed both at the site of stimulation and at distant “functionally connected” brain sites; these brain-wide networks have been shown to be consistent with known anatomical networks^11^ and have formed the basis for a mesoscale connectome project in macaque monkeys.^10^ Note that the intensities used in these studies are typically lower (three to four pulse trains, 0.1 to $0.3\;J/{\mathrm{cm}}^{2}$), directly delivered to the brain, and lead to quite focal columnar-scale stimulation. Here, we positioned the fiber with a 45 deg angle via a cover glass through a certain thickness of the cortex (depth we used in two-photon imaging). The actual intensity delivered to the neurons we observed is probably much lower. Thus, we suggest that there is sufficient excitatory neuron-driven activation (from the positive response period), to produce the hemodynamic signals seen robustly in BOLD fMRI, forming the basis for pyramidal cell mediated activation of functionally connected sites in fMRI studies.

## Supplementary Material



## Data Availability

Raw images and data are not publicly available at this time but may be obtained from the authors upon reasonable request.
