# Supplementary material for: Two-photon imaging of excitatory and inhibitory neural response to infrared neural stimulation
Source: Neurophotonics. 2024 May 24;11(2):025003. doi: 10.1117/1.NPh.11.2.025003 (PMC11125280; doi:10.1117/1.NPh.11.2.025003)
Supplement: Supplementary file 1 [file NPh_011_025003_SD001.docx]

**Two-photon imaging of excitatory and inhibitory neural response to infrared neural stimulation**

Peng Fu^1*^, Yin Liu^1,5*^, Liang Zhu^1,2^, Mengqi Wang^1^, Yuan Yu^1^, Fen Yang^1^, Weijie Zhang^2^, Hequn Zhang^1^, Shy Shoham^4^, Anna Wang Roe^1,2,3†^, Wang Xi^1,2,3†^

*^1^Interdisciplinary Institute of Neuroscience and Technology (ZIINT), the Second Affiliated Hospital, School of Medicine, Zhejiang University, Hangzhou, 310020, China*

*^2^Key Laboratory of Biomedical Engineering of Ministry of Education, College of Biomedical Engineering and Instrument Science, Zhejiang University, Hangzhou, 310027, China.*

*^3^MOE Frontier Science Center for Brain Research and Brain Machine Integration, Zhejiang University, Hangzhou, 310058, China*

*^4^Department of Ophthalmology and Tech4Health and Neuroscience Institutes, NYU Langone Health, New York, NY 10016, USA*

*^5^Laboratory for Neuro- and Psychophysiology, Department of Neurosciences, KU Leuven Medical School, Leuven, 3000, Belgium*

* These authors contributed equally to this work as co-first authors.

† Corresponding author. Email: annawang@zju.edu.cn; xw333@zju.edu.cn.

Appendix A. Supplementary data

Supplementary Table 1 and Supplementary Figures 1-7.

**Table S1. Correlation values for ‘Illuminated-region’ and ‘Weakly illuminated-region’ neurons in hSyn and mDlx anesthetized mice.**

|  | hSyn | mDlx |
| --- | --- | --- |
| Illuminated-region neurons (n = 140/88 for hSyn/mDlx) | | |
| 0.16 J/cm^2^ | 0.04 ± 0.007 | 0.01 ± 0.011 |
| 0.29 J/cm^2^ | 0.09 ± 0.008 | 0.03 ± 0.013 |
| 0.42 J/cm^2^ | 0.18 ± 0.010 | -0.12 ± 0.015 |
| 0.50 J/cm^2^ | 0.19 ± 0.012 | -0.08 ± 0.015 |
| 0.59 J/cm^2^ | 0.11 ± 0.017 | -0.04 ± 0.013 |
| 0.68 J/cm^2^ | 0.17 ± 0.014 | -0.16 ± 0.021 |
| 0.76 J/cm^2^ | 0.25 ± 0.020 | -0.12 ± 0.024 |
| Weakly illuminated-region neurons (n = 59/55 for hSyn/mDlx) | | |
| 0.16 J/cm^2^ | 0.02 ± 0.012 | 0.04 ± 0.013 |
| 0.29 J/cm^2^ | 0.07 ± 0.014 | 0.03 ± 0.014 |
| 0.42 J/cm^2^ | 0.14 ± 0.017 | -0.08 ± 0.017 |
| 0.50 J/cm^2^ | 0.15 ± 0.016 | -0.05 ± 0.016 |
| 0.59 J/cm^2^ | 0.06 ± 0.024 | -0.01 ± 0.016 |
| 0.68 J/cm^2^ | 0.13 ± 0.021 | -0.11 ± 0.027 |
| 0.76 J/cm^2^ | 0.17 ± 0.029 | -0.09 ± 0.027 |


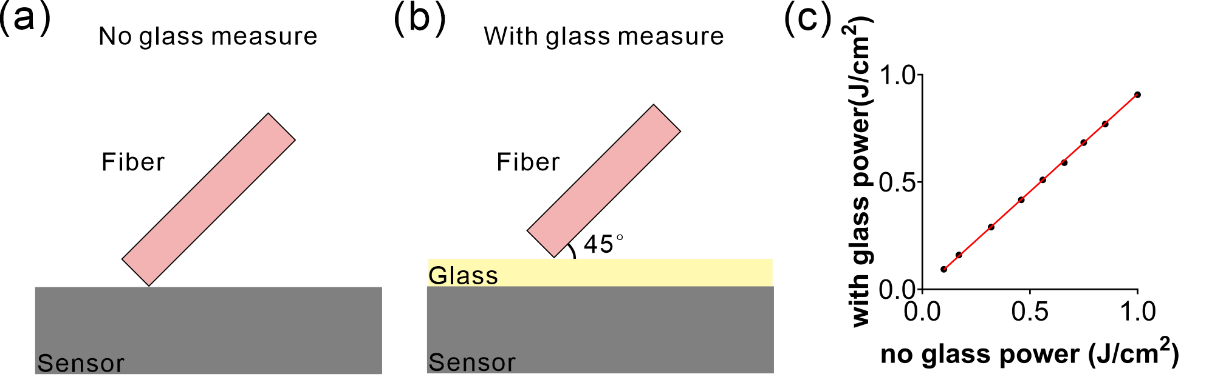


**Fig. S1.** **Correction of the applied laser energy levels for the glass.**

(a) Setup for laser energy measure with no glass at the angle of 45 degrees. (b) Setup for laser energy measure with glass at the angle of 45 degrees. (c) Fitting curve of the correction with glass (red curve, Y = 0.91×X+0.0007, R^2^ = 0.99, p < 0.0001). Data represents mean ± SEM.


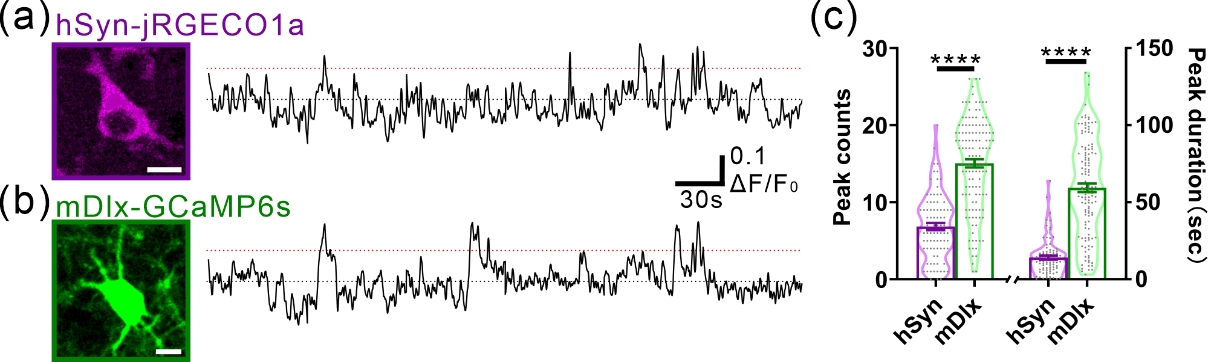


**Fig. S2. Spontaneous calcium activity of hSyn and mDlx neurons.**

(a) Example two-photon in vivo image and consecutive spontaneous activity time course of a hSyn-jRGECO1a labeled neuron. The red dashed line represents the 10% activity threshold value. (b) Example two-photon in vivo image and consecutive spontaneous activity time course of an mDlx-GCaMP6s labeled neuron. (c) Statistical comparisons of peak counts (hSyn: 6.84 ± 0.47; mDlx: 15.05 ± 0.53) and duration (hSyn: 14.07 ± 1.31 s; mDlx: 59.39 ± 2.77 s) of the spontaneous activity between hSyn neurons (n = 83 neurons in 2 awake mice) and mDlx neurons (n = 129 neurons in 2 awake mice) (peak counts, p < 0.0001; peak duration, p < 0.0001). Scale bar, 10 μm; Mann-Whitney test; data represents mean ± SEM.


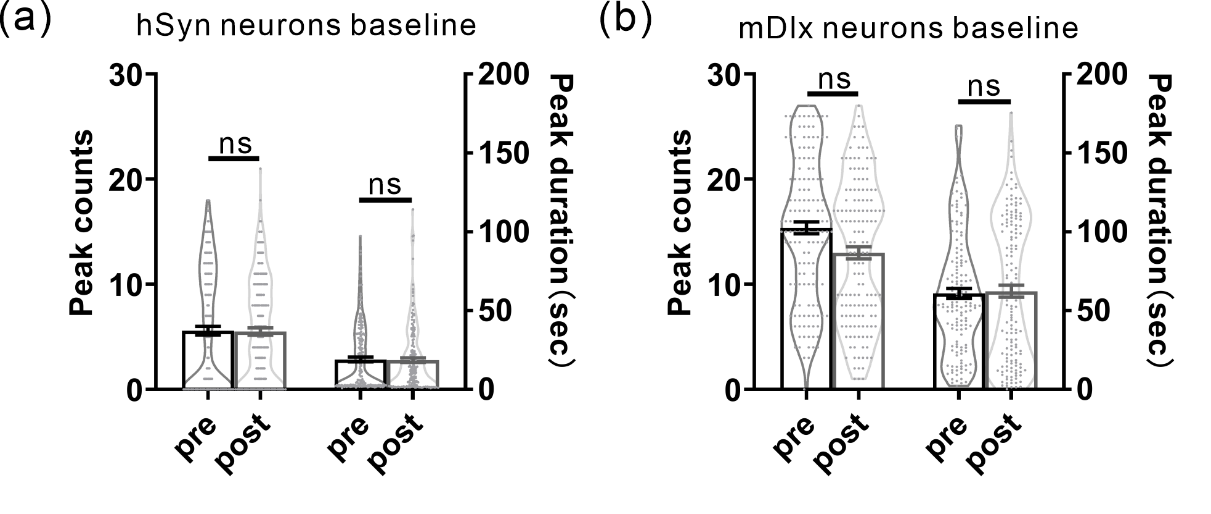


**Fig. S3. No damaging effect on neuronal spontaneous calcium activity post INS stimulation.**

(a) Statistical comparisons of peak counts (pre: 5.59 ± 0.41; post: 5.51 ± 0.35) and duration (pre: 18.97 ± 1.49 s; post: 18.61 ± 1.46 s) of the spontaneous calcium activity between pre-INS and post-INS in same hSyn neuron population (n = 199 neurons in 3 anesthetized mice) (peak counts, p = 0.5180; peak duration, p = 0.6488). (b) Statistical comparisons of peak counts (pre: 15.37 ± 0.57; post: 13.00 ± 0.57) and duration (pre: 60.87 ± 3.22 s; post: 62.27 ± 3.82 s) of the spontaneous calcium activity between pre-INS and post-INS in same mDlx neuron population (n = 143 neurons in 3 anesthetized mice) (peak counts, p = 0.0805; peak duration, p = 0.7083). Wilcoxon test; data represents mean ± SEM.


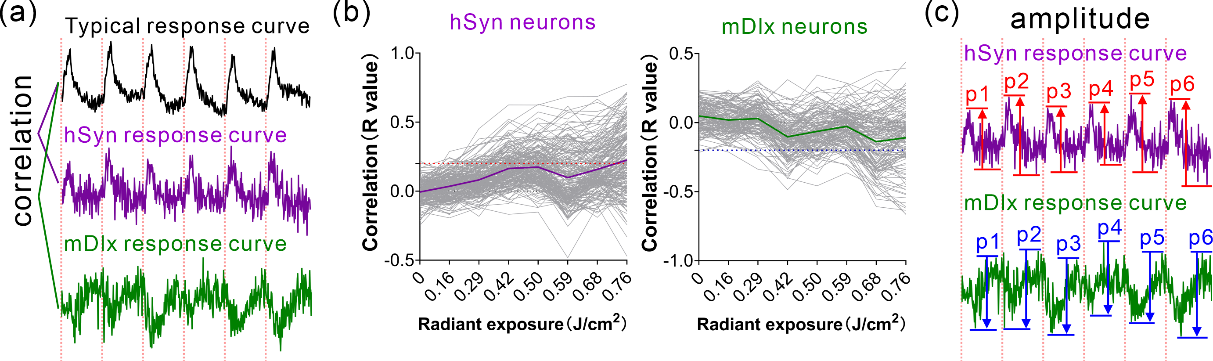


**Fig. S4. Calculation example for the correlation and amplitude value.**

(a) The correlation value is defined as the correlation between the typical response curve (the averaged INS response at 0.76 J/cm^2^) and every trial-averaged response curve for each neuron. (b) The threshold of the correlation value for positive and negative responses (n = 199 hSyn neurons and n = 143 mDlx neurons). (c) The amplitude value is defined as the average of six peaks during six pulse trains (p1, p2, p3, p4, p5, p6) for each neuron.


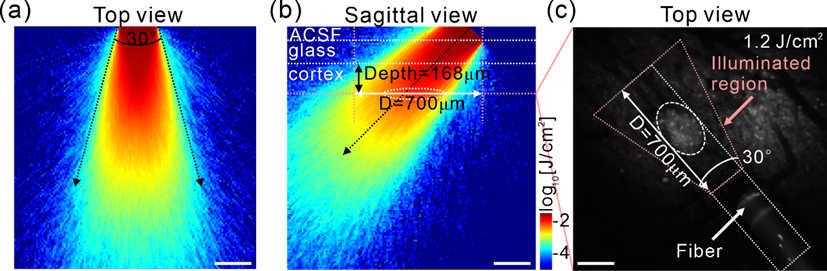


**Fig. S5. The definition of the** **illuminated region based on Monte Carlo simulation.**

(a-b) Multi-layer Monte Carlo simulation for infrared light energy. The first layer is ACSF. The second layer is the glass. The third layer is the cortex (imaging depth below cortex: 168 ± 10 μm, from n = 9 mice FOVs). The fiber is at the 45° angle. Scale bar, 200 μm. (c) At an averaged structure image, a higher radiant exposure (1.2 J/cm^2^) is used to guide the center irradiation region and combined with Monte Carlo simulation to define the illuminated region (pink region). Scale bar, 200 μm.


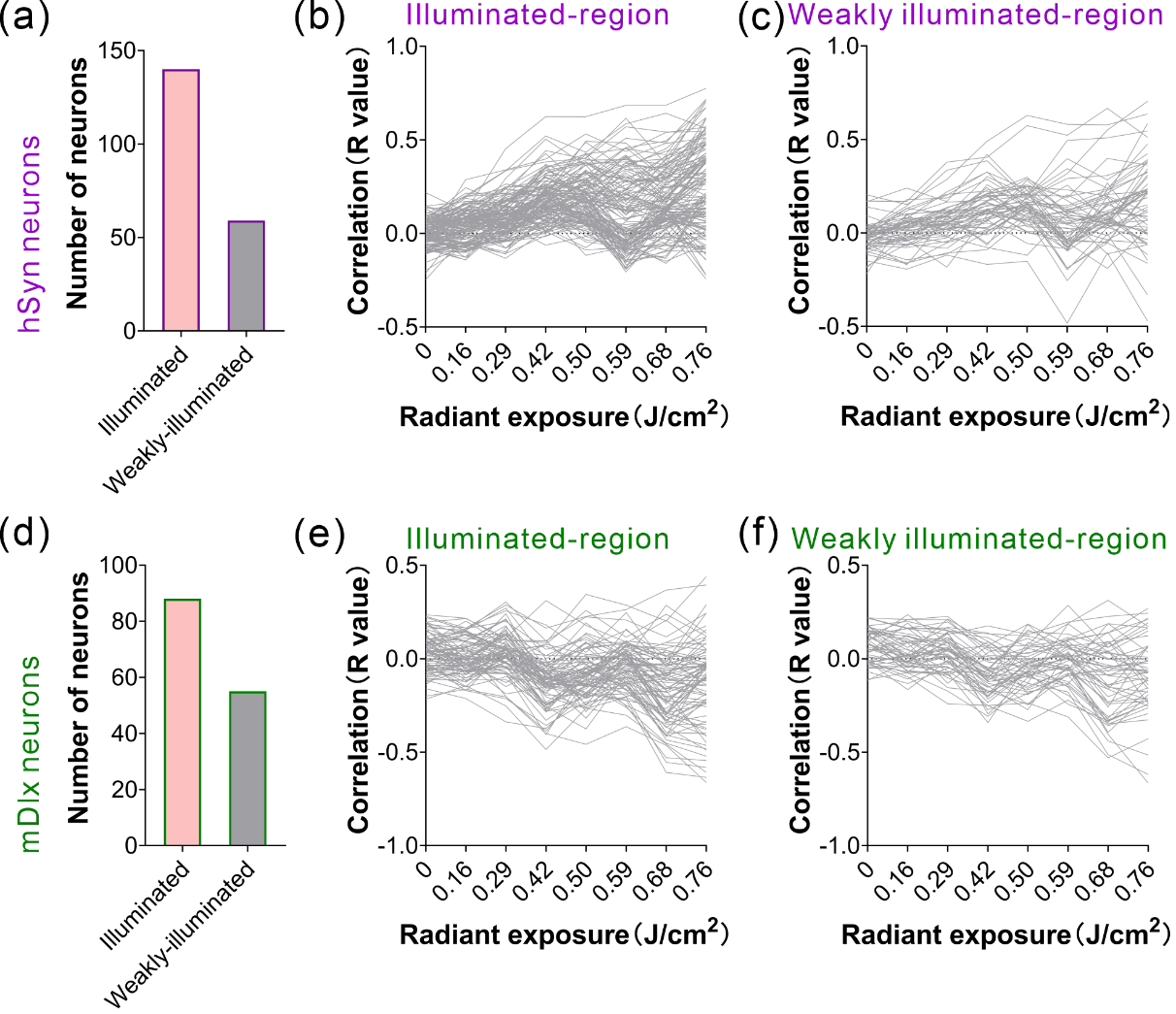


**Fig. S6. Correlation of INS-induced calcium activity of Illuminated- and Weakly illuminated-region neurons in different subtypes in anesthetized mice.**

(a-c) Correlation value of hSyn Illuminated- and Weakly illuminated-region neurons, respectively (n = 140/59 Illuminated-/Weakly illuminated-region neurons). (d-f) Correlation value of mDlx Illuminated- and Weakly illuminated-region neurons, respectively (n = 88/55 Illuminated-/Weakly illuminated-region neurons). Data in Table S1.


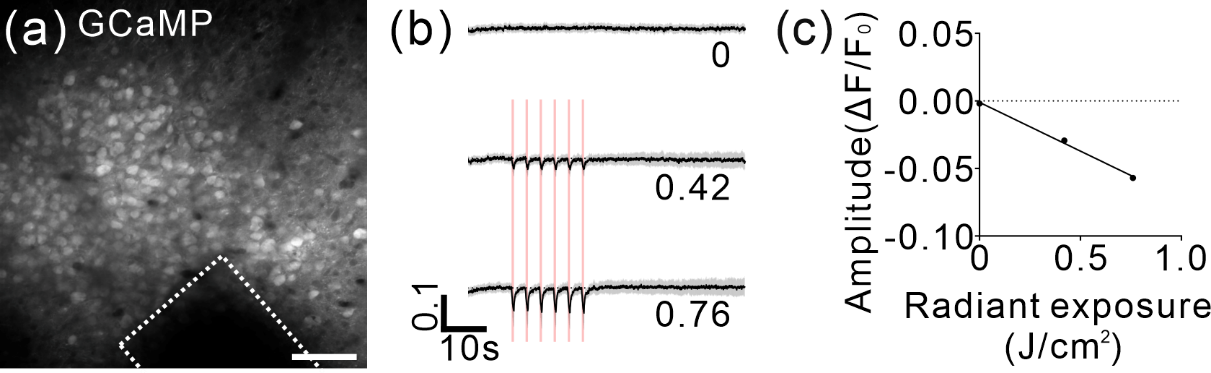


**Fig. S7. Calibration of GCaMP fluorescent changes in dead state.**

(a) hSyn-GCaMP6s expression in mouse somatosensory cortex labels neurons in dead state. The white dotted box indicates the INS fiber tip position. Scale bar, 100 μm. (b) Averaged relative fluorescence change of GCaMP6s-labeled neurons induced by INS across two intensities. Timecourse curve was mean ± SD. (c) Relative fluorescence change of GCaMP6s induced by INS as a function of the INS laser intensity (mean ± SEM, 0 J/cm^2^: -0.002 ± 0.001, 0.42 J/cm^2^: -0.029 ± 0.001, 0.76 J/cm^2^: -0.057 ± 0.001; Y = -0.07×X-0.001, R^2^ = 0.99, p = 0.0411).
